# Elevated Plasma Progastrin-Releasing Peptide Levels Associated With the Diagnosis of Pulmonary Carcinoid Tumors: A Report of Two Cases

**DOI:** 10.7759/cureus.79601

**Published:** 2025-02-24

**Authors:** Takashi Yamaoka, Kiichiro Ninomiya, Taku Noumi, Chiaki Matsumoto, Keisuke Sugimoto

**Affiliations:** 1 Department of Respiratory Medicine, Japanese Red Cross Kobe Hospital, Kobe, JPN; 2 Department of Allergy and Respiratory Medicine, Okayama University Hospital, Okayama, JPN

**Keywords:** biopsy, case report, progrp, pulmonary carcinoid, tumor marker

## Abstract

Pulmonary carcinoids are the type of thoracic malignant tumors classified as neuroendocrine tumors. Case 1 involved a 59-year-old woman with a nodular shadow in the left upper pulmonary field observed on chest radiography. Chest computed tomography (CT) scan revealed a nodule in the left upper lobe and a soft tissue tumor near the left third rib. Given her elevated plasma progastrin-releasing peptide (ProGRP) level, a CT-guided biopsy was performed, confirming a diagnosis of pulmonary carcinoid with solitary bone metastasis. She subsequently underwent partial resection of the left upper lobe and the rib tumor. Case 2 involved a 67-year-old woman referred to our hospital after a chest CT scan revealed a nodule in the left lingular region. Her plasma ProGRP level was elevated, and a CT-guided biopsy confirmed a diagnosis of pulmonary carcinoid. As no lymph node metastases were detected, she underwent segmental resection of the left upper lobe. In both cases, plasma ProGRP levels decreased following surgical resection. These findings suggest that plasma ProGRP levels may serve as a useful diagnostic marker for pulmonary carcinoids.

## Introduction

Pulmonary carcinoids are classified as neuroendocrine tumors alongside small-cell lung cancer and large-cell neuroendocrine carcinoma in the fifth edition of the World Health Organization (WHO) classification of thoracic tumors [1]. Pulmonary carcinoids are rare tumors that account for 1-2% of thoracic tumors, classified into typical carcinoids and atypical carcinoids according to their mitoses, which are indolent but potentially metastatic in nature [2]. Among neuroendocrine tumors, plasma progastrin-releasing peptide (ProGRP) (upper limit of the normal range: 81 pg/mL) is routinely used as a specific tumor marker for small-cell lung cancer in clinical practice [3]. However, its role in diagnosing pulmonary carcinoids remains unclear. Here, we report two cases where elevated plasma ProGRP levels contributed to the diagnosis of pulmonary carcinoids.

## Case presentation

Case 1

**Figure 1:**
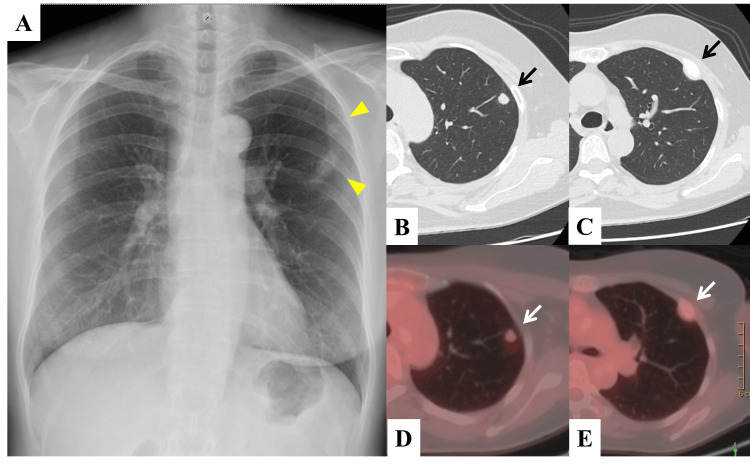
Clinical images of Case 1 (A) Chest radiograph showing two well-defined masses in the left upper pulmonary field (two arrowheads). (B and C) Chest computed tomography (CT) showing a mass measuring 9 x 7 mm in size in the left upper lobe S1+2 (arrow) and a soft tissue lesion in the left third rib (arrow). (D and E) An 18F-fluorodeoxyglucose (FDG)-positron emission tomography (PET)/CT showing accumulation in each lesion (SUVmax=1.66 and 1.96) (white arrows). SUVmax: maximum standardized uptake value

A 59-year-old woman with no history of smoking was asymptomatic; however, a chest radiograph taken during a medical checkup two months prior revealed abnormal shadows in the left upper pulmonary field. Physical examination showed no abnormalities, and her Eastern Cooperative Oncology Group (ECOG) performance status was 0. Blood tests revealed no renal impairment (blood urea nitrogen (BUN): 10.1 mg/dL; creatine (Cr): 0.55 mg/dL). Tumor marker analysis showed an elevated plasma ProGRP level of 271 pg/mL, whereas carcinoembryonic antigen (CEA) and cytokeratin-19 fragment (CYFRA) were within normal limits at 1.3 ng/mL and 0.5 ng/mL, respectively. Chest radiography (Figure 1A) revealed two nodular shadows in the left upper pulmonary field, which were slightly larger than those observed three years earlier. Chest computed tomography (CT) scan showed a nodule with mildly lobulated margins, measuring approximately 9 × 7 mm, in the left upper lobe S1+2 (Figure 1B), and a soft tissue tumor in the left third rib (Figure 1C). The latter exhibited mild accumulation on 18F-fluorodeoxyglucose (FDG) positron emission tomography (PET)/CT (SUVmax = 1.66 and 1.96, respectively) (Figures 1D-1E). A CT-guided biopsy of both tumors was performed. No obvious atypical cells were detected as the lung lesion specimen was insufficient; however, clusters of atypical cells with enlarged nuclei were identified in the bone lesion. Immunostaining revealed positivity for synaptophysin.

**Figure 2:**
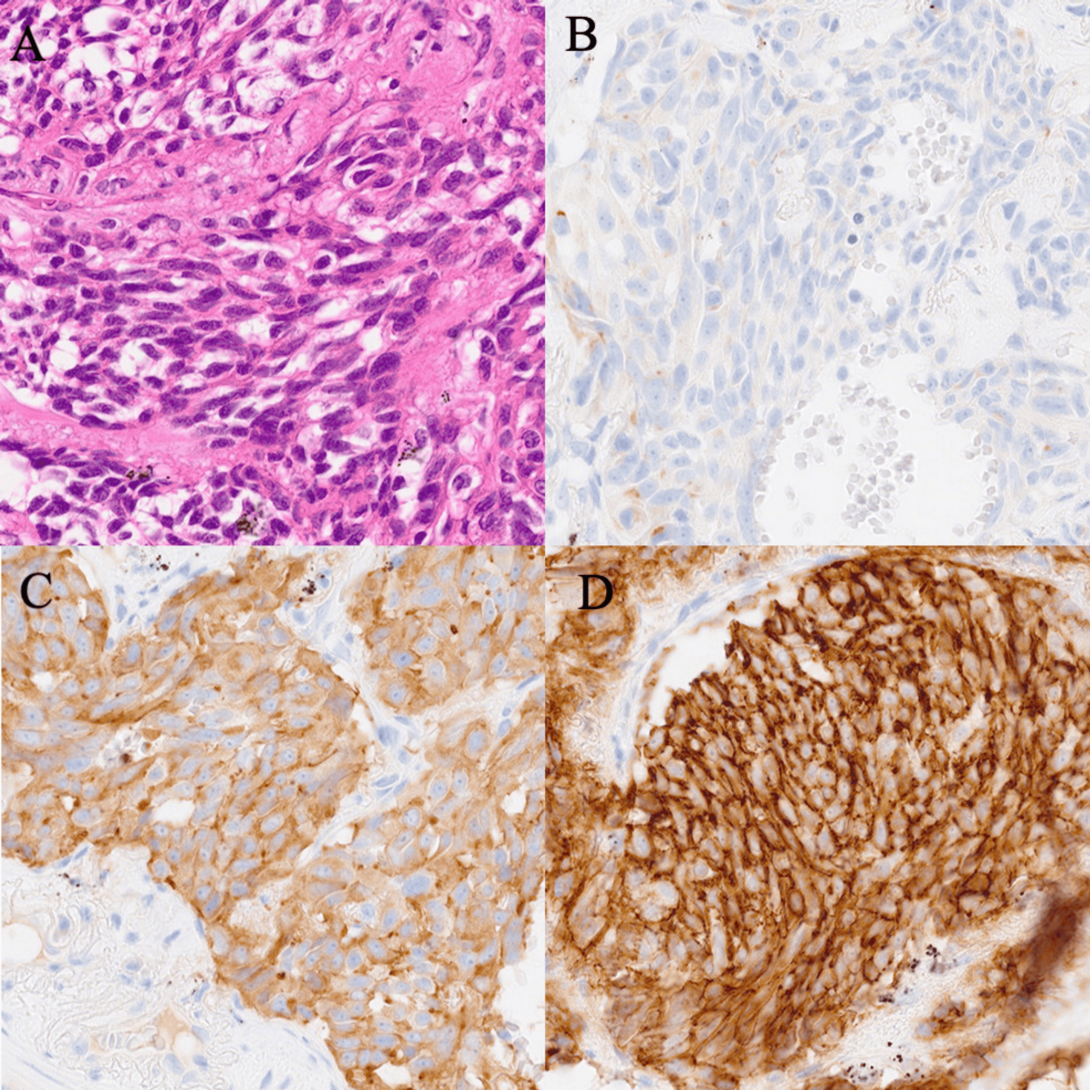
Pathological images of Case 1 (A) Hematoxylin and eosin staining of the resected specimen showing uniform tumor cells with round nuclei. No necrosis was observed, and six mitoses per 2 mm^2^ were noted. (B-D). Tumor cells tested positive for chromogranin A, synaptophysin, and CD56. All images are shown at x400.

Based on these findings, pulmonary carcinoid with bone metastasis (clinical stage IVA, T1aN0M1b) was suspected. As the tumor was localized, partial resection of the lung tumor and resection of the bone tumor were performed for a definitive diagnosis. Histopathological examination of the resected lung and bone specimens showed atypical carcinoids; thus, the diagnosis of atypical carcinoids with bone metastasis was confirmed (Figure 2). Postoperatively, plasma ProGRP levels decreased from 271 to 64.2 pg/mL. Given the advanced stage of the disease, the patient underwent four cycles of chemotherapy with cisplatin and etoposide. She has been relapse-free for 14 months, and her plasma ProGRP levels remain within the normal range.

Case 2

A 67-year-old woman was asymptomatic; however, an abnormal nodule was detected on chest CT during pulmonary cancer screening. Her medical history included bronchial asthma, hepatitis C, and postoperative appendicitis. She had no history of smoking. Physical examination revealed no abnormalities, and her ECOG performance status score was 0. Blood tests showed no renal impairment (BUN: 10.9 mg/dL; Cr: 0.76 mg/dL). Tumor marker analysis revealed an elevated plasma ProGRP level of 268 pg/mL, while CEA and CYFRA levels were within normal limits at 4.7 ng/mL and 1.1 ng/mL, respectively.

**Figure 3:**
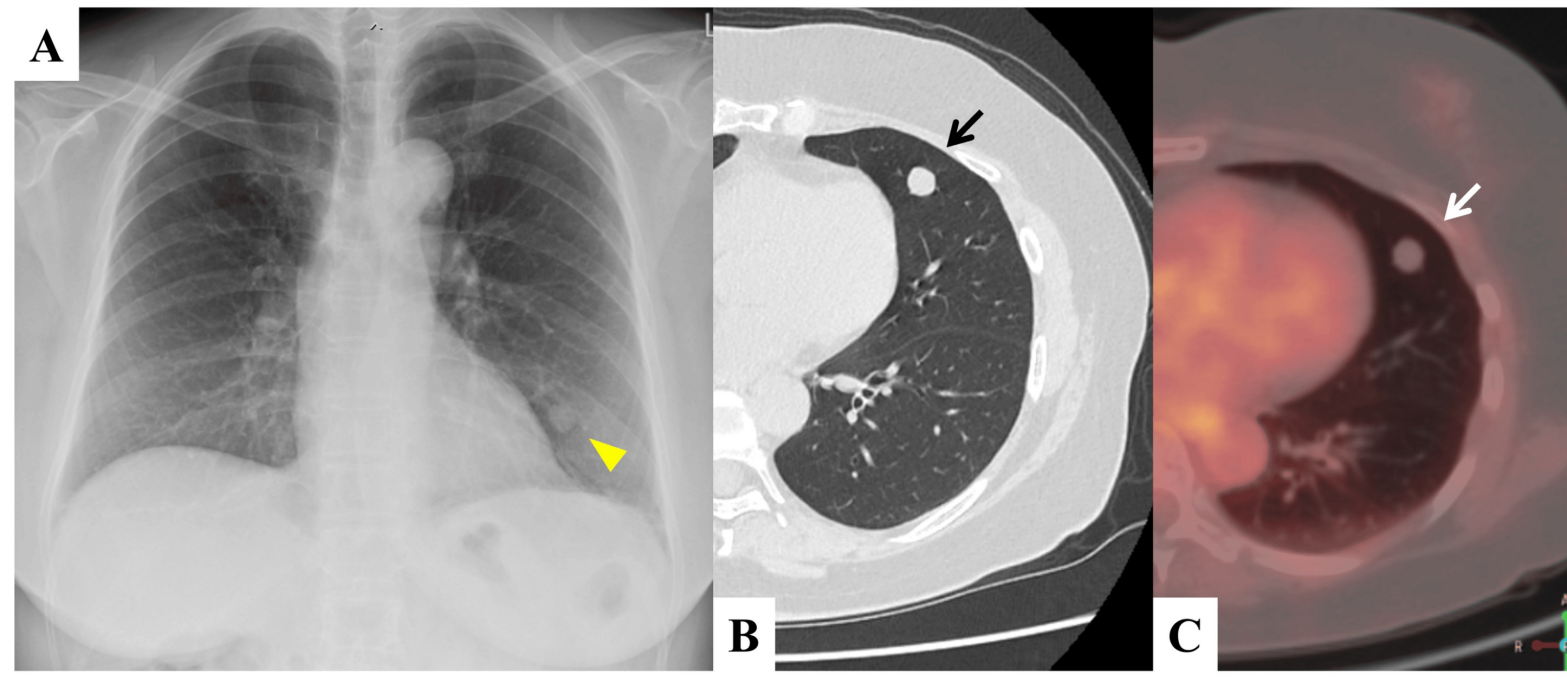
Clinical images of Case 2 (A) Chest radiograph showing a well-defined mass in the left lower pulmonary field (yellow arrowhead). (B) Chest CT showing a mass measuring 11 mm in size in the left middle lobe S5 (black arrow). (C) An 18F-FDG-PET/CT scan showing no accumulation (SUVmax=0.90) (white arrow). SUVmax: maximum standardized uptake value

**Figure 4:**
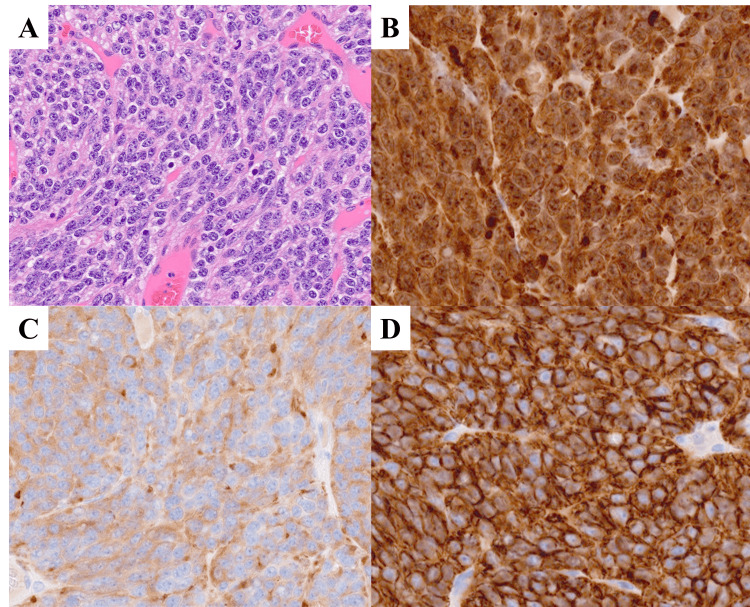
Pathological images of Case 2 (A) Hematoxylin and eosin staining of the resected specimen showing uniform tumor cells with round nuclei. No necrosis or mitosis was observed. (B-D) Tumor cells tested positive for chromogranin A, synaptophysin, and CD56. All images are shown at x400.

Chest radiography and CT showed an 11 × 10 mm nodule in S5 of the left lingual region with well-defined margins (Figures 3A-3B). An 18F-FDG PET/CT showed slight accumulation (SUVmax = 0.90) (Figure 3C). Despite the low SUVmax value, a CT-guided biopsy was performed due to the elevated plasma ProGRP level. Pathological examination confirmed a pulmonary carcinoid, leading to a diagnosis of clinical stage IA2 (T1bN0M0). The patient subsequently underwent radical treatment, including resection of the left lingual region and lymph node dissection (ND1). Postoperative pathology confirmed the tumor as a typical carcinoid (Figure 4).

Following surgery, the plasma ProGRP level decreased slightly from 268 to 186 pg/mL. The patient has remained relapse-free for eight months, and there has been no rise in ProGRP during monitoring.

## Discussion

Pulmonary carcinoids are classified as neuroendocrine tumors, along with large-cell neuroendocrine carcinoma and small-cell lung cancer. They are rare, accounting for only 1-2% of all thoracic malignancies. Morphologically, they are classified into two types: typical carcinoids, which have less than two mitoses per 2 mm^2^ and do not show necrosis, and atypical carcinoids, which have between 2 and 10 mitoses per 2 mm^2^ [1]. The 10-year survival rates for clinical stage IV cancer are 58.8% for typical carcinoids and 18.5% for atypical carcinoids [4], which are more favorable prognoses than those for small-cell lung cancer; however, despite their relatively better prognosis, pulmonary carcinoids remain potentially fatal. As these cases progress, they may be accompanied by symptoms such as hemoptysis or carcinoid syndrome, but this is not always the case [2]. Therefore, early detection is crucial; however, distinguishing pulmonary carcinoids from benign tumors remains challenging, with biopsy being the only definitive diagnostic method. The cases presented here demonstrate the potential of plasma ProGRP level measurement for early detection. These findings may serve as a valuable reference for future diagnosis and management of pulmonary carcinoids.

Currently, no tumor marker can clearly differentiate pulmonary carcinoids. However, as a type of neuroendocrine tumor, the tumor marker used for small-cell lung cancer may be measured. Plasma ProGRP levels in patients with pulmonary carcinoids are significantly lower than those in patients with small-cell lung cancer or mixed small-cell and non-small-cell lung cancer [5]. Conversely, another report showed that plasma ProGRP levels effectively distinguished pulmonary carcinoids from non-small-cell lung cancer or benign disease, with a sensitivity of 60.9% and a specificity of 89.3% [6]. Furthermore, because the plasma ProGRP level correlates with the Ki-67 index and the number of dividing cells in pulmonary carcinoids [7], higher levels may indicate more advanced cases. In contrast, CEA and other tumor markers cannot be used to distinguish pulmonary carcinoids from other thoracic malignancies [8]. Moreover, distinguishing between benign nodules and carcinoids using chest CT and other imaging modalities remains challenging [9]. Therefore, even when imaging findings suggest a benign lung tumor, measuring tumor markers, including plasma ProGRP, may provide additional diagnostic value.

Case 1 involved an advanced atypical carcinoid with bone metastasis, and all lesions were resected for a definitive diagnosis. In the case of solitary metastasis of non-small-cell lung cancer without lymph node metastasis, there is a good prognosis [10,11], and resection is an option. Chemotherapies such as cytotoxic anticancer agents, octreotide, and everolimus were considered for subsequent treatment. Although evidence for pulmonary carcinoids is limited, a retrospective study reported that the combination of etoposide and platinum agents was effective in patients with atypical carcinoids [12]; therefore, we chose this regimen for treatment. Case 2 was a typical early-stage carcinoid, for which surgical resection was the preferred approach. The effectiveness of sublobar resection for pulmonary carcinoids has not been demonstrated in prospective trials. However, multivariate analysis of the two database studies indicated that sublobar resection is not inferior to lobectomy in terms of survival [13,14]. In particular, sublobar resection is considered a viable alternative treatment for pulmonary carcinoids with a tumor diameter of 3 cm or less and no lymph node metastasis [14].

This study has some limitations. As we reported only two cases, it is not possible to fully investigate issues such as the cutoff value for ProGRP levels or the optimal follow-up protocol in pulmonary carcinoid. As there are no clear reports that link plasma ProGRP levels to prognosis in pulmonary carcinoid, further investigation is needed to determine whether treatment decisions based on plasma ProGRP levels are appropriate. However, as there is a correlation between Ki-67, which is related to cell proliferation, and plasma ProGRP levels [7], it may be important to evaluate plasma ProGRP levels over time to determine whether additional treatment, such as chemotherapy is required. Furthermore, since plasma ProGRP levels are easily affected by factors such as renal function and assay variability, their evaluation can sometimes be difficult.

## Conclusions

We report the clinical courses of two cases of pulmonary carcinoids in which elevated ProGRP levels were instrumental for diagnosis. Although imaging diagnosis is important, in cases where a benign lung tumor is suspected, it may be beneficial to measure tumor markers including ProGRP, and then consider histopathological evaluation. Further accumulation of cases is necessary for the optimal evaluation method and monitoring of plasma ProGRP levels for pulmonary carcinoids.
